# The impact of lung function case‐finding tests on smoking behaviour: A nested randomised trial within a case‐finding cohort

**DOI:** 10.1002/hsr2.41

**Published:** 2018-05-18

**Authors:** Sarah J. Ronaldson, Lisa Dyson, Laura Clark, Catherine E. Hewitt, David J. Torgerson, Brendan G. Cooper, Matt Kearney, William Laughey, Raghu Raghunath, Lisa Steele, Rebecca Rhodes, Joy Adamson, on behalf of the DOC study team

**Affiliations:** ^1^ York Trials Unit, Department of Health Sciences University of York York UK; ^2^ Lung Function and Sleep Queen Elizabeth Hospital Birmingham Birmingham UK; ^3^ University of Birmingham Birmingham UK; ^4^ NHS England and Public Health England London UK; ^5^ Hull York Medical School York UK; ^6^ James Alexander Family Practice Hull UK; ^7^ Escrick Surgery York UK; ^8^ Institute of Health and Society Newcastle University Newcastle upon Tyne UK

**Keywords:** COPD, lung function tests, randomised controlled trial, respiratory questionnaire, smoking cessation, spirometry

## Abstract

**Rationale, aims, and objectives:**

Increasing awareness of people's lung health through the use of lung function tests or symptom‐based questionnaires is a potential method to aid smoking cessation. We investigated the impact of case‐finding lung function tests for chronic obstructive pulmonary disease on smoking behaviour.

**Methods:**

Our trial used a novel waiting list randomised controlled trial design, nested within a case‐finding cohort study. The cohort comprised current smokers aged 35 years or more, from general practices in Yorkshire and Humberside, who were randomised to receive lung function tests (spirometry, microspirometry, peak flow meter measurement, and a WheezoMeter) and case‐finding questionnaires either immediately (“tests now”) or later (“waiting list” control). Outcome measures included self‐reported smoking cessation and number of cigarettes smoked at follow‐up (at 2, 3, or 6 months after randomisation, depending on study site), with 409 participants included in the primary analysis.

**Results:**

Six hundred seventy‐four participants were randomised using stratified block randomisation to the 2 groups (340 to “tests now” and 334 to “waiting list”), with 409 included in the primary analysis (194 in “tests now” and 215 in “waiting list” groups). Smoking cessation at follow‐up was very similar across groups (8.8% in the “tests now” group, compared with 9.2% in the “waiting list” group). Completing case‐finding lung function tests did not significantly impact smoking cessation (OR 1.00, 95% CI, 0.57‐1.77, adjusting for age, sex, baseline number of cigarettes smoked, and study site). A sensitivity analysis, assuming that participants with missing data were still smoking, gave similar results (OR 0.86, 95% CI, 0.47‐1.56). Analysis of the number of cigarettes smoked at follow‐up using negative binomial regression adjusting for the same factors above gave an incidence rate ratio of 0.95 (95% CI, 0.88‐1.03).

**Conclusions:**

There is no evidence from this trial of an effect of lung function tests on smoking cessation among a population of smokers aged 35 years or over. Indeed, when assuming that those with missing data were smokers, a slightly lower odds of smoking cessation was observed in the “test now” group compared with the “waiting list” group.

## 1 INTRODUCTION

Tobacco use is estimated to result in approximately 7 million deaths worldwide per year (including exposure to second‐hand smoke),^1^ with smoking being the United Kingdom's primary cause of preventable death and disease,^2^ accounting for an annual death toll of approximately 79 000 deaths.^2^ It is one of the main risk factors for vascular and respiratory disease, with approximately a third of all deaths from respiratory disease in England attributable to the consumption of cigarettes.^3^ Smoking is the most commonly cited reason for the respiratory disorder chronic obstructive pulmonary disease (COPD)^4^, with COPD being one of the most prevalent causes of morbidity and mortality worldwide,^5^ at an increasing prevalence of 10% globally among individuals aged 40 or more.^6^, ^7^ While an estimated 3 million people in the United Kingdom (UK) have COPD,^8^ 2 million have not been diagnosed with the disease.^9^

The total cost to society in England, which includes the cost to the UK National Health Service (NHS), of treating diseases caused by smoking and lost productivity due to premature deaths, smoking breaks, and absenteeism, is estimated to be £2.9 billion a year.^10^ The net ingredient cost of all prescription items used to help people stop smoking in England was £28.5 million in 2015/2016,^11^ with an evaluation of the NHS Stop Smoking Services effectiveness finding validated quit rates of 53% at 4 weeks and 15% at 1 year.^12^ Because of the high expense of such services, it is extremely valuable to explore different methods to aid smoking cessation. One such method is to increase awareness of people's lung health via use of lung function tests or symptom‐based questionnaires. Through communication about lung health and early diagnosis of COPD, smoking quit rates could be improved.

There is a paucity of good quality research evidence in this area, and the findings are currently inconclusive.^13^, ^14^, ^15^ Trials have been set up to consider the effectiveness of complex interventions around lung health, including spirometry, smoking cessation advice, and personalised feedback of lung health,^16^, ^17^, ^18^, ^19^ with the results not yet available.

This trial tested the hypothesis that having case‐finding tests for COPD (ie, tests to identify whether participants had COPD or not, where the tests comprised lung function tests and symptom questionnaires) changed self‐reported smoking behaviour among general practice patients who were smokers compared with not having the case‐finding tests.

## 2 METHODS

The primary purpose of the case‐finding cohort (DOC) study that this trial was nested within was to determine the optimal pathway to identify individuals with COPD in primary care to enhance early identification and management. The inclusion of the nested waiting list randomised controlled trial (RCT) design also allowed us to assess whether undertaking the lung function tests had an effect on smoking behaviour (the focus of this paper) and will be referred to throughout as the “trial,” as opposed to the “DOC study” or “study,” which relates to the overarching cohort study. The ISRCTN registration for the study reflects these objectives, with the primary outcome listed there relating to the case‐finding element, which has been published elsewhere,^20^ and the smoking trial outcomes being listed as secondary outcomes. The full details of the waiting list study design are available elsewhere.^21^ In brief, all participants received the same suite of case‐finding tools; however, the timing of when participants received the tests was randomly allocated in a 1:1 ratio: Those in the “tests now” (intervention) group were invited to receive their tests straight away, whereas those in the “waiting list” (control) group received the tests a few months later. The primary outcome of the trial was smoking cessation and the secondary outcome was the number of cigarettes smoked.

The patient population were smokers aged 35 and over, who were identified from 11 surgeries across 3 general practice groups in Yorkshire and Humberside and invited to participate in the DOC study. Participants were enrolled in the study using database recruitment (ie, patients at participating practices who met eligibility criteria were sent a recruitment pack through the post), and opportunistic recruitment by general practitioners (GPs) and nurses at face‐to‐face consultations. The recruitment pack given to potential participants contained an invitation letter, consent form, and a patient information sheet, which provided a detailed explanation of the study. Participants were recruited to the study on a staggered basis, where recruitment occurred over a period of time, specifically over the first half of the study, ie, over the first 6 months.^21^ Almost all participants from the DOC case‐finding cohort were entered into the trial. Individuals with a current diagnosis of COPD were included in the study. Individuals were excluded from the study if they had cognitive impairment. Once participants had been recruited, they were randomised to 1 of the 2 groups: Half were randomly allocated to receive the lung function tests straight away (the intervention group, ie, “tests now” group), and the remaining half received the tests between 2 and 6 months later, in the latter half of the trial (the control group, ie, “waiting list” group).^21^ The “tests now” intervention group had, therefore, received the “diagnostic tests intervention” by the time that smoking outcome was assessed, whereas the “waiting list” control group had not received the tests by the time of the smoking outcome assessment.^21^

At the case‐finding appointment, participants undertook a series of lung function assessments, comprising clinical history (including cough, wheeze, allergies, and asthma), WheezoMeter (KarmelSonix, Haifa, Israel) (normal breathing and deep breathing), peak flow meter, microspirometry (MS01 Micro spirometer, Care Fusion, Basingstoke, UK), relaxed vital capacity and forced vital capacity pre‐bronchodilator spirometry (EasyOn PC) (ndd Medical, Zurich, Switzerland), and symptom questionnaire—in that order. Participants with abnormal results on the spirometry were administered salbutamol via a metered dose inhaler and spacer (2‐4 separate puffs of 100 mcg) prior to conducting a third WheezoMeter reading (normal breathing) and post‐bronchodilator spirometry. Appointments could last up to 90 minutes and were led by respiratory specialist nurses in primary care. Interpretation of results was fed back to participants following usual practice, for reporting lung health and/or smoking cessation advice. The control group received usual care including smoking cessation advice. The smoking cessation advice for both groups typically involved participants being offered a stop smoking programme lasting 6 to 8 weeks, either on a one‐to‐one basis or with group support, with or without medication which could have comprised nicotine or nonnicotine products.

Prior to conducting the assessments, participants were screened against core clinical criteria, namely, pregnancy, unstable angina, pulmonary embolism or an aneurysm, pneumothorax, and unexplained haemoptysis. This screening took place initially before randomisation and was later repeated close to the appointment to reconfirm eligibility. Participants with a positive screen for any of these criteria were excluded from the intervention but kept in the trial follow‐up. After randomisation, participants were also screened for myocardial infarction or stroke; chest, abdominal, vascular, detached retina/eye, ear or brain surgery, and pacemaker implant, all within the last 12 weeks, or a current ear, chest, or gastrointestinal infection. Those screening positive were deferred for a later assessment. Changes were made to the eligibility criteria after the trial commenced. Specifically, the exclusion criteria were increased to ensure maximisation of patient safety, with all additional criteria shown in the above list.

The hypothesis concerning the effect of the case‐finding tests on smoking behaviour was evaluated by comparing the smoking habits of participants who had already undertaken the case‐finding tests (ie, participants in the “tests now” intervention group) with the smoking habits of those who have not yet undergone the case‐finding tests (ie, the “waiting list” control group).^21^ Participants' smoking habits data were obtained using a follow‐up questionnaire that was sent to all participants 2 to 6 months (dependent on site) after the month that they were allocated to their group. Hence, participants in the “waiting list” control group had not yet received their tests at this point in time.

The primary outcome measure was self‐reported smoking cessation as collected via the follow‐up questionnaire. Participants were asked “Do you currently smoke?”; those who responded “no” were classified as having stopped smoking. The number of cigarettes smoked (including hand rolled tobacco) was a secondary outcome. Participants were asked “How many cigarettes do you smoke per day?” and if they smoked hand rolled cigarettes, “How much tobacco do you use a week?,” where participants could record this in ounces or grams. To convert hand rolled tobacco into a number of cigarettes, the following calculations were undertaken: (ounces × 40) divided by 7 and ((grams × 0.04) × 40) divided by 7. Smoking behaviour was collected at baseline and at follow‐up. Follow‐up occurred at 2, 3, or 6 months after randomisation, dependent on study site.

Stratified block randomisation was utilised to assign participants to the 2 groups. We used permuted blocks with sizes from 2 to 76, which depended upon how many participants were waiting to be randomised at any given time and were stratified by practice. The randomisation sequence was concealed using York Trials Unit's secure randomised system, which was accessed by computer, and the sequence generated by an independent data manager.

There was no formal sample size calculation for this trial, as the primary purpose of the overall DOC cohort study, of which the trial was a component of, was to determine the diagnostic accuracy of different approaches for the diagnosis of COPD. The RCT element reported here was a study “add‐on,” which took advantage of the logistical delay of being able to see all the participants.

Participants, clinicians, investigators, and evaluators were not blind to the participants' group allocation because of the nature of the trial design and analysis. Allocation was concealed from the recruiting investigator.

### 2.1 Statistical analysis

No interim analysis was planned or conducted. Smoking cessation at follow‐up was compared between the 2 randomised groups using logistic regression, adjusting for the prognostic variables: age, sex, and baseline smoking pack years. A cluster term (GP practice) was also included in the model to account for variation in follow‐up time. Given the high attrition rate, a sensitivity analysis was conducted on the primary outcome, in which participants with missing data were assumed to have remained smokers and the analysis described above repeated. The number of cigarettes smoked at follow‐up was also compared between the randomised groups using negative binomial regression to account for overdispersion, adjusting for the same prognostic variables and clustering within GP practice. Analyses were prespecified and undertaken on an intention to treat basis (ie, analysed according to the groups to which participants were randomised), with all outcomes reported. Stata release 12^22^ was used for all analyses.

### 2.2 Ethical approval

The research protocol and accompanying study documents were approved by Newcastle and North Tyneside 2 Research Ethics Committee (REC reference number: 10/H0907/37). Written informed consent was obtained from all participants prior to entering into the study.

## 3 RESULTS

**Figure 1:**
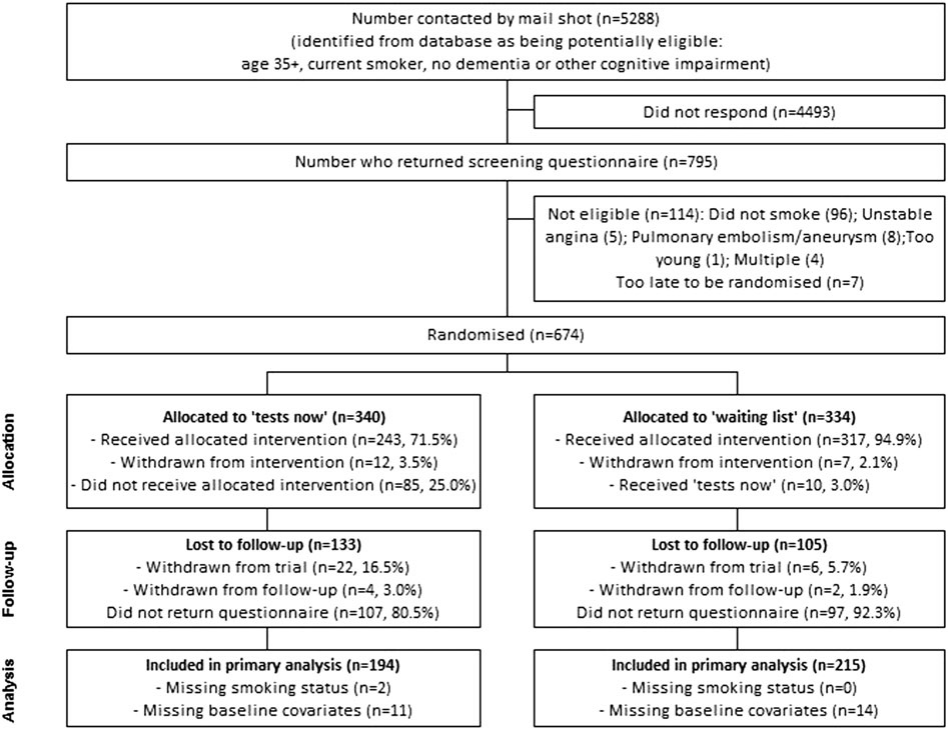
CONSORT diagram of participant flow through the DOC study

The CONSORT flow chart is shown in Figure 1. Six hundred seventy‐four participants were recruited from general practices (primary care) in the York and Hull areas of England, between June 2011 and February 2013, and were randomised in the smoking trial component, with 340 (50.4%) allocated to the “tests now” group and 334 (49.6%) to the “waiting list” group. Of the 674 participants, 441 (n = 244 “tests now,” n = 197 “waiting list”) attended the lung function tests and/or returned case‐finding questionnaires, and 434 provided follow‐up data (between December 2011 and April 2013), with 409 included in the primary analysis (Figure 1). Follow‐up data were available for 60.9% (207/340) in the “tests now” (intervention) group and for 68.6% (229/334) in the “waiting list” (control) group; ie, 133 and 105 were lost to follow‐up in the “tests now” and “waiting list” groups, respectively; follow‐up was less complete for the intervention group. The most common reasons for full withdrawal were that participants changed their mind about taking part/were no longer interested and that they were no longer smoking or had experienced a change in their health status since originally consenting. Apart from the timing of the tests, ie, the intervention group receiving the tests straight away versus those waiting to receive them (control group), the 2 groups were treated equally.

A total of 3 adverse events were encountered over the duration of the study (2 [1.0%] in the “tests now” group and 1 [0.5%] in the “waiting list” group), with all, assessed by the investigators, as being unrelated (epileptic episode, cancer diagnosis) or possibly related to the study but expected (feeling light‐headed).

### 3.1 Participant characteristics

In Table 1, we show baseline characteristics by randomised group, ie, for the “tests now” and “waiting list” groups. We present baseline data both as randomised and as analysed to assess whether or not attrition may have introduced selection bias. All baseline characteristics were comparable across randomised groups both in the randomised and analysed groups. In the analysed population, the “waiting list” group was slightly younger, weighed less, and had a lower body mass index (BMI) and fewer qualifications compared with the “tests now” group. The mean age of participants was 54 years, with a range from 35 to 82 years. The mean number of cigarettes smoked per day was 15.7, and the mean age at which participants started smoking regularly was 17. The mean BMI was 26.2, which is categorised as overweight. There were equal proportions of male and female participants. Just under half of participants left school at 16 with no qualifications, and nearly a third left school at 16 with some qualifications. Approximately 10% of participants left school at 18 with some qualifications and a further 13% went on to higher or further education.

**Table 1:** Baseline characteristics by randomised arm

	Randomised (N = 674)				Analysed (N = 409)			
	Tests Now (N = 340)		Waiting List (N = 334)		Tests Now (N = 194)		Waiting List (N = 215)	
	Mean	SD	Mean	SD	Mean	SD	Mean	SD
Age (mean)	53.5	10.9	53.4	10.9	54.4	10.5	53.6	10.5
(range)	35‐82		35‐82		36‐82		35‐80	
Number cigarettes smoked per day	15.9	8.8	16.2	9.9	15.7	8.8	15.6	9.5
Age started smoking regularly	16.4	4.7	17.4	6.2	16.8	5.5	17.4	6.2
Height, cm	168.9	10.2	169.0	9.9	169.2	10.3	168.8	10.2
Weight, kg	76.8	17.4	73.7	15.9	77.2	18.2	73.5	15.5
BMI	26.8	5.1	25.7	4.7	26.8	4.8	25.8	5.0
	N	%	N	%	N	%	N	%
Sex								
Female	172	50.6	161	48.6	96	49.5	104	48.4
Male	168	49.4	170	51.4	98	50.5	111	51.6
Highest level of educational qualification								
Left school at 16, with no qualifications	155	47.1	143	43.7	96	50.8	88	41.7
Left school at 16, with some qualifications	101	30.7	110	33.6	51	27.0	77	36.5
Left school at 18, with some qualifications	32	9.7	30	9.2	18	9.5	18	8.5
Higher education, eg, BSc and BA	35	10.6	38	11.6	21	11.1	23	10.9
Further higher education, eg, MSc and PhD	6	1.8	6	1.8	3	1.6	5	2.4

### 3.2 Analysis of smoking cessation

We included 409 participants in our primary analysis, representing those who had follow‐up data available on smoking behaviour and prognostic variables. At follow‐up, 18 participants out of 205 (8.8%) had stopped smoking in the “tests now” group, and 21 out of 229 (9.2%) had stopped smoking in the “waiting list” group (Table 2). A logistic regression of smoking cessation at follow‐up on randomised group, adjusted for age, sex, baseline number of cigarettes smoked, and clustering by study site, gave an odds ratio (OR) of 1.00 (95% confidence interval [CI], 0.57‐1.77, *P* = .997). The wide confidence interval indicates the uncertainty of the effect estimate and its poor precision. The OR of 1.00 indicates the same odds of smoking cessation in the “tests now” group compared with the “waiting list” group. Thus, there was no evidence of an effect of lung function tests on smoking cessation.

**Table 2:** Smoking cessation at follow‐up by randomised arm and assuming all participants with missing outcome data are still smoking

	Tests Now		Waiting List		Total	
	N	%	N	%	N	%
**All available data**						
Number who have stopped smoking at follow‐up	18	8.8	21	9.2	39	9.0
Number who returned follow‐up questionnaire	205		229		434	
COPD diagnosis	50	24.0	26	11.4	76	17.5
**Those included in the primary analysis**						
Number who have stopped smoking at follow‐up	17	8.8	19	8.8	36	8.8
Number who returned follow‐up questionnaire	194		215		409	
COPD diagnosis	32	16.5	18	8.4	50	12.2

*Assuming all missing are still smoking*						
**All available data**						
Number who have stopped smoking at follow‐up	18	5.3	21	6.3	39	5.8
Total number of participants	340		334		674	
**Those included in the primary analysis**						
Number who have stopped smoking at follow‐up	17	5.2	19	5.9	36	5.6
Total number of participants	326		320		646	

### 3.3 Secondary analyses

Assuming that all participants with missing outcome data were still smoking, there was a 5.3% smoking cessation rate in the “tests now” group and a 6.3% cessation rate in the “waiting list” group (Table 2). A logistic regression of smoking cessation on randomised group, adjusted for age, sex, baseline number of cigarettes smoked, and clustering by study site, gave an OR of 0.86 (95% CI, 0.47‐1.56; *P* = .623). This indicates a lower odds of smoking cessation in the “tests now” group compared with the “waiting list” group, although the difference was not statistically significant.

### 3.4 Smoking cessation by recruitment method

Most of the participants in the trial were recruited via database recruitment, with only 8% recruited opportunistically by a nurse or GP during a face‐to‐face consultation. When undertaking an exploratory analysis according to method of recruitment, 8.5% quit in the database recruitment group compared with 14.3% in the opportunistic group (Table 3).

**Table 3:** Smoking cessation by recruitment method

Recruitment method:	Database		Opportunistic		Total	
	N	%	N	%	N	%
**All available data**						
Number who have stopped smoking at follow‐up	34	8.5	5	14.3	39	9.0
Number who returned questionnaire	399		35		434	
**Those included in the primary analysis**						
Number who have stopped smoking at follow‐up	31	8.2	5	15.2	36	8.8
Number who returned questionnaire	376		33		409	

### 3.5 Analysis of number of cigarettes smoked

At follow‐up, the average number of cigarettes smoked was similar in both groups; “tests now” 13.8 (SD 8.9, with median 15 [min 0 to max 60]) and “waiting list” 13.8 (SD 8.2, with median 15 [min 0 to max 40]). An analysis of the number of cigarettes smoked at follow‐up using negative binomial regression on randomised group, adjusted for the same prognostic variables and study site, gave an incidence rate ratio of 0.95 (95% CI, 0.88‐1.03; *P* = .241). This indicates a higher number of cigarettes smoked at follow‐up in the “tests now” group compared with the “waiting list” group, although this difference was not statistically significant. Thus, there was no evidence of an effect of lung function tests on the number of cigarettes smoked at follow‐up.

## 4 DISCUSSION

Having case‐finding tests for lung function (intervention group) did not change smoking behaviour compared with not having the tests (control group) among general practice patients who were self‐reported smokers. Indeed, participants in the intervention group (“tests now”) were equally likely to stop smoking than those in the control group (“waiting list”). A sensitivity analysis assuming that all participants with missing outcomes were still smoking at follow‐up did not impact on the overall result, but under this assumption, participants in the intervention group were less likely to stop smoking than those in the control group, although these findings were not statistically significant. Based on this assumption, the overall quit rate across both arms of the trial (5.8%) was similar to the background quit rates observed in the control arm for comparable populations.^23^ An exploration of the impact of recruitment method on smoking cessation found that a higher cessation rate was highlighted among those recruited opportunistically compared with those recruited through a database. This difference could have arisen because those recruited opportunistically were keen to quit and in contact with practice staff more frequently. For interpreting the similarity in quit rates found by our trial, it is useful to note that 29% (97/340) in the “tests now” group did not receive the tests and 3% (10/334) in the “waiting list” group received their tests before intended. In addition, 50 (12.2%) participants included in the analysis were found to have COPD (32 (16.5%) in the “tests now” group and 18 (8.4%) in the “waiting list” group). The higher proportion of COPD participants in the intervention (“tests now”) group could be a potential reason for greater loss to follow‐up in this group compared with the “waiting list” group, as individuals might have felt judged if they were still found to be smoking at follow‐up. Of the 32 found to have COPD in the “tests now” group, 3 (9.4%) quit, and in the “waiting list” group, 5 of the 18 (2.8%) quit (Table 2). When comparing the overall quit rates for participants with COPD to those with normal lung function, the trial found a lower quit rate for those with normal lung function (7.8%; 28/359) compared with participants with a COPD diagnosis (16.0%; 8/50). This is in line with what may be expected, in that participants with normal lung function were less inclined to quit.

### 4.1 Strengths and weaknesses

We did not exclude patients who had already been diagnosed with COPD; hence, patients who were already aware of their airflow obstruction could have responded differently to the lung function tests/smoking cessation advice compared with those with previously undetected obstruction. However, randomisation would have addressed this issue in that the proportion of patients with or without prior knowledge of obstruction would be balanced between groups. A further limitation relates to the issue of attrition: We were able to analyse smoking behaviour data for 61% of participants; hence, for 39% of participants, data were missing. However, examination of the baseline characteristics of those participants who had data available for analysis did not suggest any major selection bias due to attrition, and the assumption of all those with missing data being smokers did not materially change our findings. It is worthwhile noting that there is potential bias arising from learning effects, in that participants who had their study appointments towards the end of the trial will have been seen by nurses who became more experienced by this time than at the beginning of the trial. Because of practicalities of the study appointments at our different sites, the follow‐up time point used was not fixed for all participants; instead, the follow‐up point varied between 2 months for some participants, and 6 months for others, dependent on the study site. Therefore, the quit rate was evaluated at different time points for different participants: 72 at 2 months, 202 at 3 months, and 135 at 6 months, although the analyses took account of this.

The smoking data (smoking status and number of cigarettes smoked) analysed in our trial were self‐reported by participants rather than an independent evaluation using cotinine testing, which would have been a more objective measure of point prevalence.^24^, ^25^ Perhaps surprisingly, the loss to follow‐up was greater in the “tests now” group (39%) than in the “waiting list” group (31%). A likely explanation for this differential was the low response rate and subsequent withdrawal of participants who received a letter to invite them to book a case‐finding appointment. Loss to follow‐up was subsequently reduced when participants were contacted by an alternative telephone method for the remaining test now group and all of the waiting list group.

### 4.2 Results in relation to other studies

Most of the studies that have attempted to consider the possible impact of lung health on smoking cessation (including those in the Cochrane review^13^) have been part of a complex intervention utilising different methods of communicating lung health, with or without additional smoking cessation interventions. A small, 3‐armed trial used the Fletcher curve^26^ to provide confrontational feedback of spirometric results combined with an antidepressant for smoking cessation and medium‐intensity counselling.^27^ This study found no difference in cotinine‐validated prolonged abstinence rates between the experimental group (11.2%) and the control group (11.6%) receiving an equally intensive treatment without the confrontational spirometry from weeks 5 to 52 (OR 0.96, 95% CI, 0.43‐2.18). The abstinence rate was approximately twice as high in the experimental group compared with the control group receiving standard spirometry and usual care (5.9%), but this difference was not statistically significant (OR 2.02, 95% CI, 0.63‐6.46).

Two small trials evaluated spirometry without personalised communication of lung age combined with counselling and/or nicotine replacement therapy compared with similar counselling and/or nicotine replacement therapy without spirometry.^28^, ^29^ These trials showed small and nonsignificant effects, which remained nonsignificant when pooled in the Cochrane review.^13^

In contrast, one large trial conducted in UK general practice^23^ found a significant effect of communicating lung age with routine smoking cessation advice and the offer of referral to local smoking cessation services, on smoking cessation. However, people with worse spirometric lung age were not more likely to quit than those with normal lung age. Parkes et al concluded that telling smokers their lung age significantly improves the likelihood of them quitting smoking, but the mechanism by which this intervention achieves its effect is unclear.^23^ In addition, Lin (in Quanjer and Enright) highlights that in order to establish the independent motivational effectiveness of doing spirometry screening versus not, a randomised trial in which the control arm did not receive spirometry is required.^30^ In the trial reported here, we compared the impact of being in receipt of several measures of “lung health” versus none.

An evidence review was recently conducted by NICE^31^ as part of their development of guidance on smoking interventions and services, which is soon to become available. This involved a series of systematic reviews being undertaken regarding the effectiveness and cost‐effectiveness of different interventions for smoking cessation. Of most relevance for our trial were the findings relating to whether brief advice (less than 10 min) or very brief advice (less than 30 s) from a community, health, or social care professional was found to be effective and cost‐effective. The review did not identify any published evidence relating to very brief advice interventions of less than 30 seconds; however, 2 Cochrane reviews were identified regarding brief advice of less than 10 minutes.^32^, ^33^ On pooling data from 17 studies, Stead et al^33^ found that brief advice (with or without a leaflet during a single consultation of less than 20‐min duration and up to one follow‐up visit) provided by physicians or physicians supported by other health care workers versus no advice (or usual care) increased quit rates (RR 1.66, 95% CI, 1.42‐1.94), with the authors concluding that simple advice has a small effect on quit rates. Similarly, when evaluating the effect of nurse‐delivered brief advice (single 10‐min consultation with one follow‐up visit) compared with no advice or usual care, Rice et al^32^ found an increase in quit rates (7 trials; RR 1.27, 95% CI, 0.99‐1.62), although this increase was not statistically significant.

## 5 CONCLUSIONS

There was no evidence from this trial to support the hypothesis that delivering lung function tests, as would be used in case‐finding for early identification of COPD, would have an impact on smoking behaviour. This trial adds to the existing evidence around the use of case‐finding lung function tests for smoking cessation, and the findings can be applied to individuals who currently smoke, where smoking cessation options are being explored. The heterogeneity of the interventions between previous studies limited the scope to combine the findings with the DOC study in a meta‐analysis. Findings from ongoing trials^16^, ^17^, ^18^, ^19^ may help to provide more conclusive evidence as to whether complex interventions including the individualised communication of lung age alongside education and/or nicotine replacement therapy impact on smoking cessation.

## FUNDING

This trial was funded by the Department of Health Respiratory Programme, as part of the overarching DOC study.

## CONFLICT OF INTERESTS

The authors declare that they have no conflict of interests.

## AUTHOR CONTRIBUTIONS

Conceptualisation: Joy Adamson, Lisa Dyson, Catherine Hewitt, Sarah Ronaldson, David Torgerson

Data Curation: Catherine Hewitt, Sarah Ronaldson

Formal Analysis: Catherine Hewitt, Sarah Ronaldson

Funding Acquisition: Joy Adamson, Lisa Dyson, Catherine Hewitt, Sarah Ronaldson, David Torgerson

Investigation: Rebecca Rhodes, Lisa Steele

Methodology: Joy Adamson, Brendan Cooper, Lisa Dyson, Catherine Hewitt, Matt Kearney, Sarah Ronaldson, David Torgerson

Project Administration: Laura Clark, Lisa Dyson, Sarah Ronaldson

Resources: Laura Clark, Lisa Dyson, Catherine Hewitt, Sarah Ronaldson

Supervision: Joy Adamson, Brendan Cooper, Matt Kearney, William Laughey, Raghu Raghunath, David Torgerson

Visualisation: Catherine Hewitt, Sarah Ronaldson

Writing—review and editing: Joy Adamson, Laura Clark, Brendan Cooper, Lisa Dyson, Catherine Hewitt, Matt Kearney, William Laughey, Raghu Raghunath, Rebecca Rhodes, Sarah Ronaldson, Lisa Steele, David Torgerson

Writing—original draft: Lisa Dyson, Catherine Hewitt, Sarah Ronaldson
